# Association between IL-13 +1923C/T polymorphism and asthma risk: a meta-analysis based on 26 case-control studies

**DOI:** 10.1042/BSR20160505

**Published:** 2017-01-27

**Authors:** Yueli Xu, Junjuan Li, Zhaolei Ding, Juan Li, Bin Li, Zhengang Yu, Wei Tan

**Affiliations:** 1 Postgraduate Department of Internal Medicine, Weifang Medical University, Weifang, Shandong, China; 2 Department of Respiratory Medicine, Weifang People’s Hospital, Weifang, Shandong, China

**Keywords:** Asthma, interleukin-13, meta-analysis, polymorphism

## Abstract

Asthma is a serious and hereditary respiratory disorder affecting all age groups. Interleukin-13 (IL-13) is a central regulator of allergic inflammation. The purpose of the present study was to estimate the relationship between IL-13 +1923C/T polymorphism and asthma susceptibility. Relevant case-control studies published between January 2000 and July 2016 were searched in the online databases. Review Manage (RevMan) 5.3 was used to conduct the statistical analysis. The pooled odds ratio (OR) with its 95% confidence interval (CI) was employed to calculate the strength of association. A total of 26 articles were retrieved, including 17642 asthma patients and 42402 controls. Overall, our results found that IL-13 +1923C/T polymorphism was significantly associated with increased risk of asthma under each genetic model (*P*<0.00001). Subgroup analysis by ethnicity showed that alleles and genotypes of this variant correlated with asthma among Asians and Caucasians, but only TT genotype under the homozygote model in Africans. When stratified by age group, this variant highly correlated with asthma in children and moderately in adults. Furthermore, the TT, CT and CC genotypes in asthma group were all significantly associated with increased IgE levels in sera of asthma patients when compared with controls. Our results suggested that IL-13 +1923C/T polymorphism contributed to the development of asthma. Further case-control studies with more ethnicities are still needed.

## Introduction

Asthma, a heterogeneous disease, is the most common long-term inflammatory disease of the lung airways, remaining a major cause of disability, health resource utilization and poor quality of life worldwide [1,2]. It is characterized by the presence of recurring respiratory symptoms, reversible airflow obstruction and bronchospasm [3]. Its symptoms include episodes of wheezing, chest tightness, shortness of breath and coughing, occurring a few times a day or a week depending on the single individual [4]. According to the World Health Organization, it is estimated that 235 to 330 million people currently suffer from asthma, and approximately 250000 to 345000 people die from this disease per year throughout the world [5]. The risk factors such as tobacco smoking, indoor allergens, obesity, diet, air pollution and social factors might contribute to the development of asthma [6,7]**.** In addition, an elevation of serum IgE level is considered as a potent predictor of asthma course [8]. Although great advances for the development of asthma controller therapies have been made and death rates due to asthma have reduced greatly over these years, no available therapeutic regimens can cure this disease and its burden will continue to be driven by increasing prevalence [9]. Therefore, there is an urgent need to identify some biomarkers to predict this disease and create a guideline for the therapeutic strategies.

Recent progresses in molecular biology suggest complex interactions of innate and adaptive immune cells, structural cells and their cytokines involved in the process of airway inflammation [10,11]. A growing number of evidence have shown that interleukins (ILs) are critical to mounting inflammation and immune responses [12]. ILs are a multifunctional group of immunomodulators that primarily mediate the leucocyte cross-talk, and mainly regulate the immune cell proliferation, growth, differentiation, survival, activation and functions [13–15]. The interleukin-13 (*IL-13*) gene, located on human chromosome 5q31–33, is produced by innate lymphoid cells and T-helper type 2 (Th2) cells during allergic inflammation, containing four exons and three introns and encoding an unglycosylated protein composed of 132 amino acids. The most prominent effects of IL-13 include promotion of differentiation and survival of eosinophils and mast cells, activation of fibroblasts, elevation of bronchial hyperresponsiveness and switching of B-cell antibody production from IgM to IgE [16]. Furthermore, IL-13 inhibition may be beneficial in patients who are refractory to existing therapies [17]. Studies have shown that IL-13 might play a role in human diseases such as allergic airway disease [18], fibrosis [19] and renal cell carcinoma [20]. Genetic polymorphisms in *IL-13* gene might influence its function, thus involved in the pathogenicity of asthma. One of the most studied single nucleotide polymorphisms (SNPs) was +1923C/T (rs1295686) in third intron at the intron/exon boundary. This variant was shown to be involved in the dysregulation of total IgE [21].

Several studies have identified the role of IL-13 +1923C/T polymorphism in asthma susceptibility; however, the results obtained from different geographical populations were very different. For example, Ramphul et al. [22] found that IL-13 +1923C/T locus had a significant effect predisposed to asthma in Mauritian Indian children, not in Chinese Han population. Moreover, the prevalence rates of asthma vary between countries, ranging from 1 to 18% and it is more common in developed than in developing countries [23]. Also, genetic associations with asthma can differ significantly among different ethnic populations [24,25]. Therefore, we conducted this meta-analysis to reassess the association of IL-13 +1923C/T polymorphism with asthma risk based on all the available case-control studies.

## Materials and methods

### Study identification

The electronic databases of Medline, Embase, PubMed, Chinese National Knowledge Infrastructure (CNKI) and Wanfang were comprehensively searched to retrieve relevant articles published between January 2000 and July 2016. The following medical subject heading (MeSH) terms: “asthma or asthmatic”, “cytokines or interleukin or interleukin-13 or IL-13” and “polymorphism or variant or SNP” as well as their combinations were employed as the searching keywords. The corresponding Chinese version was used in the Chinese databases. To obtain more data, we manually searched the references of related articles. Our analysis only focused on the studies that were written in English and Chinese. When the same authors or laboratories reported this issue on the same population, only the latest published full-text article was included.

### Inclusion and exclusion criteria

The included studies must meet the following criteria: (i) case-control studies evaluating the correlation of IL-13 +1923C/T polymorphism in asthma risk; (ii) patients with asthma were defined according to the Guidelines of the American Thoracic Society [26] or other diagnostic criteria [27]; controls should be unrelated ethnically matched individuals with no symptoms or history of allergy and other pulmonary diseases; (iii) genotype information in patients and controls was available to extract; and (iv) the genotype distribution in controls should be in consistence with Hardy–Weinberg equilibrium (HWE). The exclusion criteria were: (i) without the control group; (ii) conference papers or review reports; (iii) data cannot be extracted; and (iv) with duplicated data.

### Data extraction

Two of our authors independently assessed the extracted information of each included study. Any disagreement was resolved by discussion with a third author. Each item should be able to reach a final consensus. The following information was extracted from each article: the name of first author, published year, country, ethnicity, mean age, sample size, genotyping methods, genotype distribution and HWE in controls.

### Statistical analysis

The association between IL-13 +1923C/T polymorphism and asthma risk was measured by pooled odds ratio (OR) with 95% corresponding confidence intervals (CIs). The significance of the pooled OR was determined by the *Z*-test and a *P* value less than 0.05 was considered significant. The allelic model (T compared with C), homozygote model (TT compared with CC), heterozygote model (CT compared with CC), dominant model (TT + CT compared with CC) and recessive model (TT compared with CT + CC) were calculated. The *I*^2^-test and the Q-statistic test were employed to determine the between-study heterogeneity. The fixed-effect model was used when the *P*-value for the Q-test was more than 0.10 and *I*^2^ for the *I*^2^ test was <50%; otherwise, the random-effect model was used. Funnel plot was used to assess the publication bias. Analyses were performed using the software Review Manage 5.3 (Oxford, England, U.K.).

## Results

### Main characteristics of selected studies

**Figure 1:**
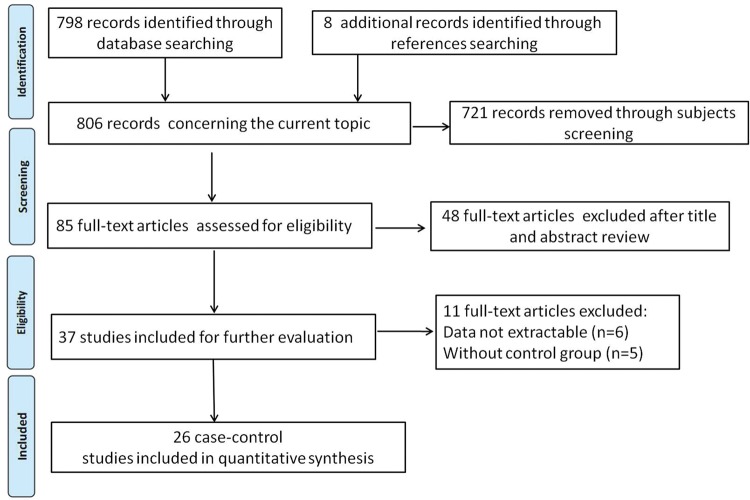
Flow chart of selection process in this meta-analysis

Figure 1 outlined the study process of selection. Briefly, we first identified 798 articles. After applying the inclusion and exclusion criteria, a total of 26 articles including 17642 asthma patients and 42402 controls were screened out. Of the 26 articles, 12 were written in Chinese [28–39] and 14 in English [40–53]. Among them, 17 were conducted in Asian populations, five in Caucasian populations and four in African populations. One article contained two study populations. The IL-13 +1923C/T polymorphism was measured by 11 different methods. The genotypes of IL-13 +1923C/T polymorphism in controls were all in accordance with HWE (*P*>0.05). Table 1 listed the main characteristics of included studies. Table 2 exhibited the distribution information of alleles and genotypes of IL-13 +1923C/T polymorphism.

**Table 1:** Main characteristics of included studies in this meta-analysis

First author	Year	Country	Ethnicity	Mean age		Sample size		Genotyping methods
				Cases	Controls	Cases	Controls	
Hákonarson, H.	2001	Iceland	Caucasian	38 (12–59)	38 (12–59)	94	94	PCR
Chen, J.Q.	2004	China	Asian	2.59 ± 1.44	2.90 ± 1.45	96	53	PCR-RFLP
Donfack, I, J.	2005	U.S.A.	Caucasian	NA	NA	126	205	LAS
Donfack, II, J.	2005	U.S.A.	African	NA	NA	205	183	LAS
Song, Z.Q.	2005	China	Asian	14–67	18–70	100	100	PCR-RFLP
Battle, N.C.	2007	U.S.A.	African	19.4 (7.3–40.9)	29.8 (8.2–41.2)	264	176	PCR-RFLP
Shi, X.H.	2008	China	Asian	34 (14–66)	18–56	48	48	PCR-RFLP
Daley, D.	2009	Australia	Caucasian	NA	NA	644	751	Illumina Bead Array System
Wang, X.H.	2009	China	Asian	39 ± 11	48 ± 12	150	160	PCR-RFLP
Li, X.N.	2010	U.S.A.	Caucasian	46.9 ± 18.4	31.4 ± 21.9	473	1892	Illumina HumanCNV370 BeadChip
Moffatt, M.F.	2010	Mixed	Caucasian	NA	NA	10365	16110	Illumina Human610 quad array
Wu, X.H.	2010	China	Asian	8.8 ± 3.2	9.2 ± 2.8	252	227	PCR-RFLP
Noguchi, E.	2011	Japan	Asian	8.0 ± 4.4	60.3 ± 14.2	938	2376	TaqMan
El-Behady, E.M.	2012	Egypt	African	9.3 ± 1.8	9.2 ± 1.6	50	30	PCR-RFLP
Yoon, D.K.	2012	Korea	Asian	52.2 ± 8.9	52.2 ± 8.9	237	16095	Afymetrix Genome-Wide Human SNP array 5.0
Jia, C.M.	2013	China	Asian	4.27 ± 2.52	4.15 ± 2.91	77	50	PCR-RFLP
Kelibiena, T.	2013	China	Asian	38.35 ± 9.17	38.12 ± 8.23	76	89	PCR-RFLP
Liu, Q.H.	2013	China	Asian	3–12	NA	384	384	TaqMan
Pu, H.P.	2013	China	Asian	5.8 ± 2.9	5.6 ± 2.6	96	96	PCR-RFLP
Wang, Y.	2014	China	Asian	3–12	3–12	435	601	SNaPshot assay
Xia, M.Q.	2014	China	Asian	6.30 ± 3.39	4.96 ± 3.61	305	200	PCR-RFLP
Xu, J.X.	2014	China	Asian	7.5 ± 8.2	8.4 ± 8.8	230	220	Sequenom
Ramphul, K.	2015	Mauritius	African	3–12	18–22	193	189	TaqMan
Xi, S.Y.	2015	China	Asian	20–65	18–66	100	100	PCR-RFLP
Li, T.X.	2016	China	Asian	5.06 (4.0–8.0)	5.00 (4.0–8.0)	652	752	SnaPshot assay
Tang, M.F.	2016	China	Asian	2–7	2–7	903	1205	TaqMan
Wang, H.L.	2016	China	Asian	8 ± 1.7	6.6 ± 2.0	173	56	PCR-RFLP

LAS, multiplex PCR and an immobilized linear array system; NA, not available; PCR-RFLP, PCR-restriction fragment length polymorphism.

**Table 2:** Distribution information of alleles and genotypes in IL-13 +1923C/T polymorphism among asthma patients and controls

First author	Cases					Controls					
	CC	CT	TT	C	T	CC	CT	TT	C	T	HWE
Hákonarson, H.	65	27	2	157	31	64	27	3	155	33	0.997
Chen, J.Q.	41	43	12	125	67	39	14	0	92	14	0.541
Donfack, I, J.	72	45	9	189	63	120	77	8	317	93	0.598
Donfack, II, J.	18	101	86	137	273	25	75	83	125	241	0.486
Song, Z.Q.	24	55	21	103	97	43	47	10	133	67	0.860
Battle, N.C.	31	117	113	179	343	21	77	72	119	221	0.998
Shi, X.H.	12	26	10	50	46	30	16	2	76	20	0.997
Daley, D.	422	199	23	1043	245	516	213	22	1245	257	0.939
Wang, X.H.	31	57	61	119	179	66	68	26	200	120	0.498
Li, X.N.	278	167	28	723	223	1247	578	67	3072	712	0.998
Moffatt, M.F.	6306	3558	501	16170	4560	10310	5156	644	25776	6444	0.984
Wu, X.H.	106	114	32	326	178	126	85	16	337	117	0.949
Noguchi, E.	387	439	112	1213	663	1125	1025	226	3275	1477	0.944
El-Behady, E.M.	20	20	10	60	40	25	5	0	55	5	0.883
Yoon, D.K.	110	99	28	319	155	7729	6768	1580	22226	9928	0.081
Jia, C.M.	22	42	13	86	68	25	22	3	72	28	0.812
Kelibiena, T.	37	26	13	100	52	66	19	4	151	27	0.274
Liu, Q.H.	174	164	46	512	256	179	169	36	527	241	0.912
Pu, H.P.	39	45	12	123	69	67	24	5	158	34	0.379
Wang, Y.	182	191	62	555	315	291	246	64	828	374	0.542
Xia, M.Q.	84	128	93	296	314	95	73	32	263	137	0.997
Xu, J.X.	150	71	9	371	89	151	62	7	364	76	0.979
Ramphul, K.	78	79	25	235	129	77	96	13	250	122	0.066
Xi, S.Y.	21	41	38	83	117	41	44	15	126	74	0.854
Li, T.X.	304	290	58	898	406	355	316	81	1026	478	0.698
Tang, M.F.	345	439	110	1129	659	512	530	150	1554	830	0.781
Wang, H.L.	38	58	77	134	212	24	23	9	71	41	0.690

### Association of IL-13 +1923C/T polymorphism in asthma risk

**Figure 2:**
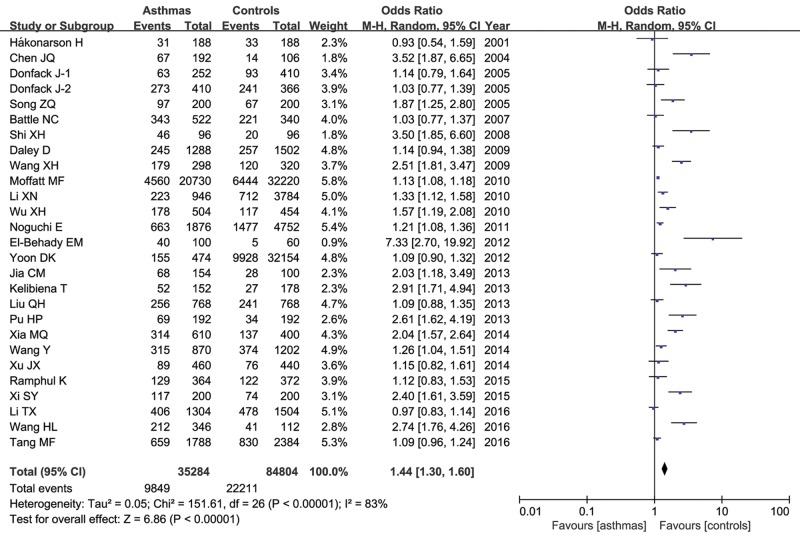
Meta-analysis of the correlation between the IL-13 +1923C/T polymorphism and asthma risk under the allelic model

Table 3 presented the genetic effect of IL-13 +1923C/T variant on asthma risk. The between-study heterogeneity was detected (*P<*0.01 and *I*^2^>50%), and the random-effect model was employed. Overall, the frequency of T allele of IL-13 +1923C/T polymorphism was found to be a little higher in asthma patients than that in controls (27.9% compared with 26.2%), and the statistical analysis demonstrated that this allele was significantly related with increased asthma susceptibility (T compared with C: OR=1.44, 95% CI= 1.30–1.60, *P*<0.00001) as shown in Figure 2. This significant association was obtained in other genetic models as well (TT compared with CC: OR=1.93, 95% CI=1.57–2.37, *P*<0.00001; CT compared with CC: OR=0.32, 95% CI=1.20–1.46, *P*<0.00001; TT + CT compared with CC: OR=1.49, 95% CI=1.32–1.68, *P*<0.00001; TT compared with CT + CC: OR=1.59, 95% CI=1.34–1.89, *P*<0.00001).

**Table 3:** Summary of the genetic effect of IL-13 +1923C/T variant on asthma risk and subgroup analyses

Groups	Comparisons	*N*	Test of association		Test of heterogeneity		
			OR (95% CI)	*P*	*I*^2^	Ph	Model
Total	T compared with C	26	1.44 (1.30, 1.60)	<0.00001	83%	<0.00001	R
	TT compared with CC		1.93 (1.57, 2.37)	<0.00001	73%	<0.00001	R
	CT compared with CC		1.32 (1.20, 1.46)	<0.00001	59%	<0.0001	R
	TT + CT compared with CC		1.49 (1.32, 1.68)	<0.00001	76%	<0.0001	R
	TT compared with CT + CC		1.59 (1.34, 1.89)	<0.00001	67%	<0.0001	R
Asian	T compared with C	17	1.66 (1.41, 1.95)	<0.00001	86%	<0.00001	R
	TT compared with CC		2.34 (1.72, 3.18)	<0.00001	79%	<0.00001	R
	CT compared with CC		1.46 (1.27, 1.69)	<0.00001	60%	0.0005	R
	TT + CT compared with CC		1.74 (1.45, 2.08)	<0.00001	78%	<0.00001	R
	TT compared with CT + CC		1.85 (1.43, 2.39)	<0.00001	73%	<0.00001	R
Caucasian	T compared with C	5	1.14 (1.09, 1.18)	<0.00001	0%	0.41	F
	TT compared with CC		1.30 (1.16, 1.46)	<0.00001	0%	0.46	F
	CT compared with CC		1.31 (1.08, 1.19)	<0.00001	0%	0.72	F
	TT+CT compared with CC		1.15 (1.10, 1.21)	<0.00001	0%	0.56	F
	TT compared with CT + CC		1.25 (1.11, 1.40)	0.0001	0%	0.52	F
African	T compared with C	4	1.32 (0.88, 1.98)	0.18	79%	0.003	R
	TT compared with CC		1.59 (1.09, 2.31)	0.02	45%	0.14	F
	CT compared with CC		1.45 (0.77, 2.74)	0.25	73%	0.01	R
	TT + CT compared with CC		1.60 (0.83, 3.08)	0.16	77%	0.005	R
	TT compared with CT + CC		1.26 (0.76, 2.09)	0.38	63%	0.04	R
Adult	T compared with C	6	1.66 (1.20, 2.31)	0.002	86%	<0.00001	R
	TT compared with CC		2.61 (1.42, 4.79)	0.002	78%	0.0004	R
	CT compared with CC		1.27 (1.10, 1.48)	0.001	42%	0.12	F
	TT + CT compared with CC		1.66 (1.18, 2.34)	0.004	77%	0.0007	R
	TT compared with CT + CC		2.19 (1.35, 3.56)	0.002	70%	0.006	R
Children	T compared with C	14	1.50 (1.27, 1.77)	<0.00001	84%	<0.00001	R
	TT compared with CC		1.90 (1.40, 2.57)	<0.0001	73%	<0.00001	R
	CT compared with CC		1.38 (1.17, 1.62)	<0.0001	64%	0.0006	R
	TT + CT compared with CC		1.56 (1.29, 1.89)	<0.00001	78%	<0.00001	R
	TT compared with CT + CC		1.59 (1.23, 2.07)	0.0004	67%	0.0002	R

F, fixed-effect model; *N*, number of included studies; R, random-effect model.

### Subgroup analysis by ethnicity and age group of the association between IL-13 +1923C/T polymorphism and asthma risk

**Figure 3:**
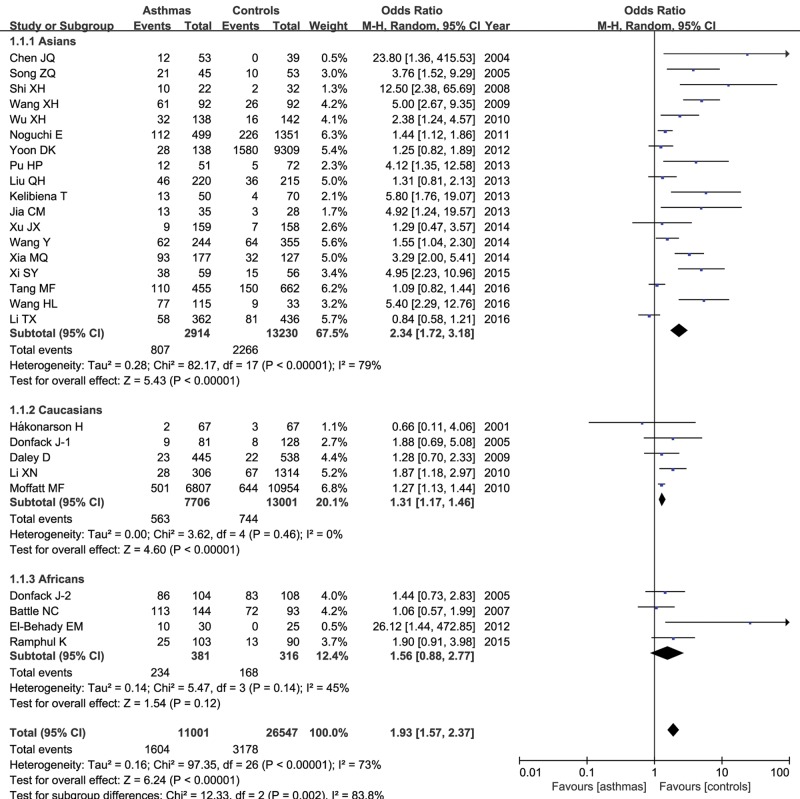
Forest plot of the relative strength of the association between IL-13 +1923C/T polymorphism and asthma risk under the homozygote model among Asians, Caucasians and Africans

In the stratified analysis by ethnicity, 17 articles including 5242 asthma patients and 22781 controls were conducted in Asians, five articles including 11702 patients and 19052 controls in Caucasians, and four articles including 698 patients and 569 controls in Africans. Overall, we found that IL-13 +1923C/T variant was associated with increased asthma susceptibility among Asians under each genetic model (T compared with C: OR=1.66, 95% CI=1.41–1.95, *P*<0.00001; TT compared with CC: OR=2.34, 95% CI=1.72–3.18, *P*<0.00001; CT compared with CC: OR=1.46, 95% CI=1.27–1.69, *P*<0.00001; TT + CT compared with CC: OR=1.74, 95% CI=1.45–2.08, *P*<0.00001; TT compared with CT + CC: OR=1.85, 95% CI=1.43–2.39, *P*<0.00001). This statistical significance was detected in Caucasians as well (T compared with C: OR=1.14, 95% CI=1.09–1.18, *P*<0.00001; TT compared with CC: OR=1.30, 95% CI=1.16–1.46, *P*<0.00001; CT compared with CC: OR=1.31, 95% CI=1.08–1.19, *P*<0.00001; TT + CT compared with CC: OR=1.15, 95% CI=1.10–1.21, *P*<0.00001; TT compared with CT + CC: OR=1.25, 95% CI=1.11–1.40, *P*=0.0001). However, only TT genotype under the homozygote model was related with increased risk of asthma in Africans (TT compared with CC: OR=1.59, 95% CI=1.09–2.31, *P*=0.02) in the fixed-effect model. Figure 3 showed the relationship between TT genotype of +1923C/T variant and asthma risk under the homozygote model among Asians, Caucasians and Africans respectively.

**Figure 4:**
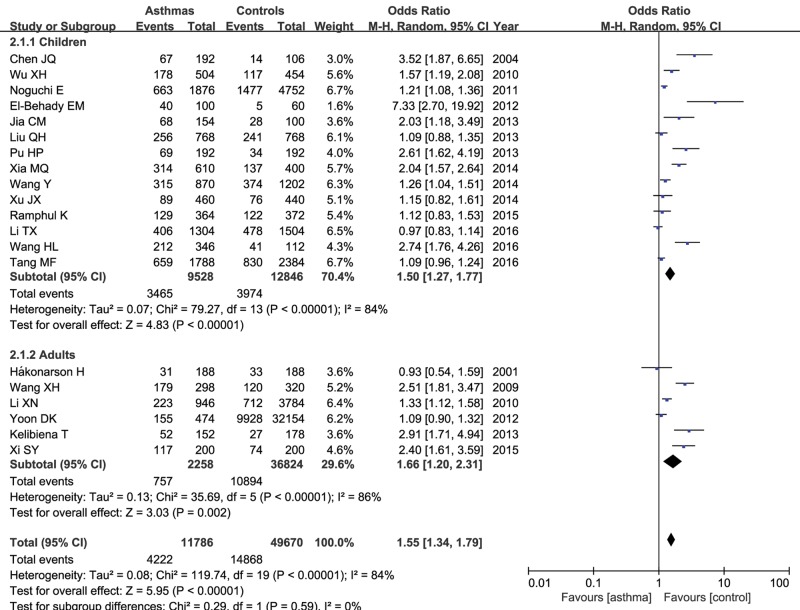
Meta-analysis of correlation of IL-13 +1923C/T polymorphism in asthma in children and adults

In the stratified analysis by age group, 14 articles containing 4764 cases and 6423 controls were performed in children (<18 years old), six articles containing 1129 cases and 18412 controls were in adults (≥18 years old) and the other six were mixed age group. Our result identified that IL-13 +1923C/T variant correlated with increased risk of asthma in both children and adult groups under each genetic model (Table 3). The significant effect was higher in children group than that in adult group. Figure 4 showed the relationship between T allele of IL-13 +1923C/T polymorphism and asthma risk in children and adults respectively.

### Association of IL-13 +1923C/T polymorphism in IgE levels (k-units/l)

**Figure 5:**
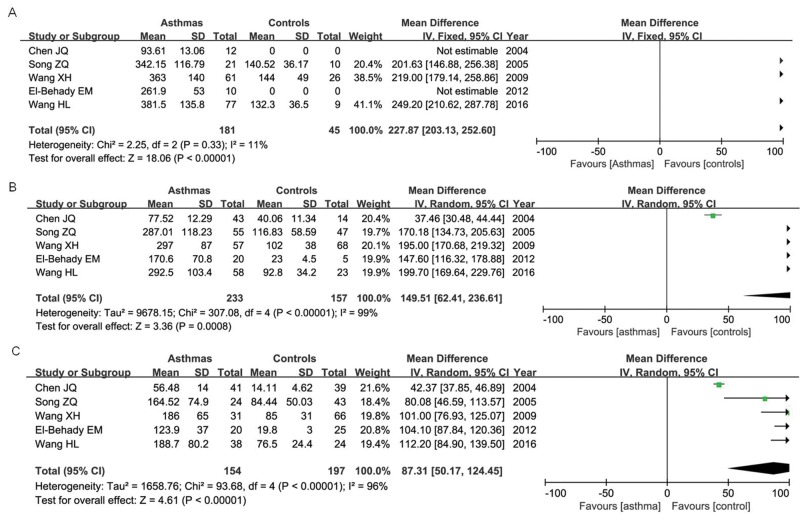
Forest plot of the association between TT (A), CT (B) and CC (C) genotypes of IL-13 +1923C/T polymorphism and IgE level (k-units/l) of patients with asthma

Eight articles reported the association between IL-13 +1923C/T polymorphism and IgE levels in serum; however, the relevant data could only be extracted from five of them, including 569 asthma patients and 399 controls. The statistical analysis found that the TT, CT and CC genotypes in asthma group were all significantly associated with increased IgE levels in serum when compared with controls as shown in Figure 5.

### Sensitivity analysis and publication bias

**Figure 6:**
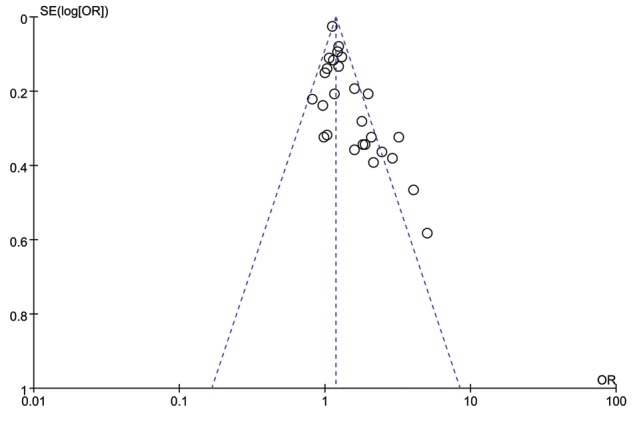
Funnel plot of IL-13 +1923C/T polymorphism in asthma risk under the heterozygote model

We omitted each particular study to verify whether our results were influenced by each included study or not. The pooled ORs were not materially altered. The funnel plot was used to evaluate the publication bias. All the plots were found to be roughly symmetrical, indicating no publication bias presented as shown in Figure 6.

## Discussion

In this meta-analysis, we retrieved a total of 26 relevant articles. Our results found that IL-13 +1923C/T polymorphism was significantly associated with increased risk of asthma under each genetic model. Subgroup analysis by ethnicity showed that the alleles and genotypes of this genetic variant correlated with asthma susceptibility among Asians and Caucasians, but only TT genotype under the homozygote model in Africans. This variant was related with increased risk of asthma in both children group and adult group under each genetic model as well. Furthermore, the TT, CT and CC genotypes in asthma group were all significantly associated with increased IgE levels in sera of asthma patients when compared with controls. Our result was consistent with previous meta-analysis conducted by Liu et al. [54], which contained ten included studies and suggested that IL-13 +1923C/T polymorphism was a risk factor for asthma.

Asthma is a hereditary disorder that is caused by a combination of intrinsic factors and environmental exposure [55]. Exposure to allergens is one of the environmental factors. In response to allergen presentation by airway DCs, T-helper lymphocytes of the adaptive immune system control many aspects of the disease through secretion of IL-4, IL-5, IL-13, IL-17 and IL-22, and these are counterbalanced by cytokines produced by Treg cells [56]. IL-13 is a key Th2 cytokine that directs many of the important features of airway inflammation and remodelling in patients with allergic asthma [57]. The IL-13 transcriptional “signature” can be used to identify individuals with “Th2 high” and “Th2 low” asthma [58]. IL-13 induces characteristic changes in mRNA [59] and miRNA [60] expression patterns in airway epithelial cells, and it induced protein periostin that is secreted basally from airway epithelial cells and can be used as a biomarker for Th2 high asthma [61]. Furthermore, sputum IL-13 levels could serve as a useful biomarker for asthma control assessment [62]. Current studies have identified that target IL-13 pathway is a promising therapeutic approach for asthma [63,64]. The association of IL-13 with asthma pathology and reduced corticosteroid sensitivity suggests a potential benefit of anti-IL-13 therapy in refractory asthma [65,66].

Asthma is primarily an inflammatory disorder of the airways associated with Th2 cell-dependent promotion of IgE production and recruitment of mast cells [67]. The elevated level of total IgE and allergy-specific IgE may function as independent risk factors for asthma [68–70]. IL-13 is known to be a key regulator in IgE synthesis. IgE production in allergic asthma patients is more dependent on IL-13 than in non-atopics, due to enhanced IL-13 production and to enhance IgE production in response to IL-13 [71]. SNPs in IL-13 were shown to be associated with allergic phenotypes in several ethnically diverse populations and might affect IgE level [72,73,76]. Allelic variation in the IL-13 gene was robustly confirmed as a contributor to the variance of IgE levels [74]. T allele of IL-13 +1923C/T was highly significant associated with total serum IgE (*P*=0.00022) [51]. Li et al. [75] found this variant was significantly associated with asthma risk in Chinese children and adults. The potential mechanism might be that: IL-13 +1923C/T variant is located in the third intron of *IL-13* gene. The +1923C was easy to be DNA methylated, thus inhibiting the transcription of *IL-13* gene; while the +1923T variant might suppress this inhibitive effect, thus promoting high expression of IL-13 and serum IgE level. In addition, other IL-13 polymorphisms might be associated with asthma risk as well. IL-13 rs20541 and rs1800925 were risk factors for asthma and rs1800925 was significantly associated with total serum IgE levels [77]. SNP rs848 in the *IL-13* gene region was significantly associated with a continuous measure of symptom severity in adult subjects with severe asthma [78].

Several limitations were presented in our meta-analysis. Firstly, the between-study heterogeneity in any genetic models was high, which might influence the result. Secondly, most of the included studies were conducted in Asian and Caucasian populations, although other ethnicities should be considered. Thirdly, different genotyping methods were used in the respective studies, which may be associated with different call rates. Lastly, the interaction of gene–gene and gene–environment should be considered.

In conclusion, our results suggested that IL-13 +1923C/T polymorphism was a risk factor for asthma susceptibility, especially in Asians and Caucasians. Future large-scale and well-designed studies with more ethnicities are still required to validate the relationship between this genetic polymorphism and asthma risk.

## Abbreviations

CI: confidence interval
HWE: Hardy–Weinberg equilibrium
IL: interleukin
IL-13: interleukin-13
OR: odds ratio
SNP: single nucleotide polymorphism
Th2: T-helper type 2
